# Artificial intelligence in medicine: A comprehensive survey of medical doctor’s perspectives in Portugal

**DOI:** 10.1371/journal.pone.0290613

**Published:** 2023-09-07

**Authors:** Ana Rita Pedro, Michelle B. Dias, Liliana Laranjo, Ana Soraia Cunha, João V. Cordeiro

**Affiliations:** 1 NOVA National School of Public Health, Public Health Research Centre, Comprehensive Health Research Center, CHRC, NOVA University Lisbon, Lisbon, Portugal; 2 NOVA National School of Public Health, Public Health Research Centre, Universidade NOVA de Lisboa, Lisbon, Portugal; 3 Westmead Applied Research Centre, Faculty of Medicine and Health, The University of Sydney, Australia; 4 CICS.NOVA Interdisciplinary Center of Social Sciences, Universidade NOVA de Lisboa, Lisbon, Portugal; University of Namibia, NAMIBIA

## Abstract

Artificial Intelligence (AI) is increasingly influential across various sectors, including healthcare, with the potential to revolutionize clinical practice. However, risks associated with AI adoption in medicine have also been identified. Despite the general understanding that AI will impact healthcare, studies that assess the perceptions of medical doctors about AI use in medicine are still scarce. We set out to survey the medical doctors licensed to practice medicine in Portugal about the impact, advantages, and disadvantages of AI adoption in clinical practice. We designed an observational, descriptive, cross-sectional study with a quantitative approach and developed an online survey which addressed the following aspects: impact on healthcare quality of the extraction and processing of health data via AI; delegation of clinical procedures on AI tools; perception of the impact of AI in clinical practice; perceived advantages of using AI in clinical practice; perceived disadvantages of using AI in clinical practice and predisposition to adopt AI in professional activity. Our sample was also subject to demographic, professional and digital use and proficiency characterization. We obtained 1013 valid, fully answered questionnaires (sample representativeness of 99%, confidence level (*p*< 0.01), for the total universe of medical doctors licensed to practice in Portugal). Our results reveal that, in general terms, the medical community surveyed is optimistic about AI use in medicine and are predisposed to adopt it while still aware of some disadvantages and challenges to AI use in healthcare. Most medical doctors surveyed are also convinced that AI should be part of medical formation. These findings contribute to facilitating the professional integration of AI in medical practice in Portugal, aiding the seamless integration of AI into clinical workflows by leveraging its perceived strengths according to healthcare professionals. This study identifies challenges such as gaps in medical curricula, which hinder the adoption of AI applications due to inadequate digital health training. Due to high professional integration in the healthcare sector, particularly within the European Union, our results are also relevant for other jurisdictions and across diverse healthcare systems.

## Introduction

The scientific foundations of Artificial Intelligence (AI) were established almost 70 years ago [1]. Depending on context, AI can be defined differently [2–7]. In summary, AI is a computer science field that develops systems capable of manipulating concepts and data, using heuristics, representing knowledge and incorporating inaccurate or incomplete data, allowing solutions, and facilitating learning [8].

AI progress to date has been described in generations: application to generic tasks, such as image, text and sound recognition, analysis, and interpretation (1^st^ generation) [7, 9–12]; software that can act autonomously, comparable to intelligent human life (2^nd^ generation) [13]; and self-aware systems, which surpass human intelligence and can be applied in any area (3^rd^ generation) [6]. Currently, the use of first-generation AI has expanded across different sectors and generative AI has been considered by some authors as an early example of 2nd generation AI [13–17].

In healthcare, AI has demonstrated its capability to assist healthcare professionals with administrative and clinical tasks, including routine work [18], diagnosis and prescription decision-making [2, 19]. Furthermore, AI has been proposed as a potential solution for significant healthcare challenges, including reducing medical errors in diagnostics, drug treatments, and surgeries, optimizing resource utilization, and improving workflows [20, 21]. Its application in performing routine and repetitive tasks shows potential for enhancing efficiency, accuracy, and impartiality in healthcare [22].

Due to its expanding reach and broad potential, some argue that implementation of AI tools can revolutionize healthcare [23], particularly given the abundance of data, complex problems, and diverse operational contexts in the health sector, including medicine [22, 24]. With the advancements in big data analytics, AI can uncover crucial information and extract knowledge that may be inaccessible even to skilled medical professionals [25]. Promising AI applications include integrating health information, user education, pandemic and epidemic prevention, medical diagnosis, and decision support models across various clinical contexts [26–36].

However, AI applications in healthcare also elicit significant technical, ethical, legal, and social challenges [20, 37–40]. Building trust and confidence in AI’s positive outcomes in medicine is essential for its acceptance and adoption by health professionals [41–43]. Therefore, evaluating healthcare professionals’ perspectives on AI’s potential and impact is essential for successful integration [42, 44–47]. Recent studies indicate that some professionals remain reticent about preparedness, resource availability, and economic implications of AI implementation [48–50]. Furthermore, education and training on AI are identified as important requirements [49]. Additionally, involving all stakeholders is deemed fundamental [46] and some medical specialists view AI as a "co-pilot" rather than an independent clinical decision-maker [49]. Connectivity issues, infrastructure gaps, and potential negative consequences also raise concerns [48, 51, 52]. In parallel, studies among medical students indicate their awareness of AI’s potential in healthcare, particularly in radiology, and their willingness to embrace it in their daily lives [45, 50, 53, 54]. They recognize the importance of including AI training in medical education, as students who received such training feel more confident in utilizing AI [55]. Both healthcare professionals and patients express skepticism and hope concerning privacy and health data protection in AI applications [52], underscoring the need to test and adapt AI systems in healthcare to meet stakeholders’ needs [56].

Despite the growing usage of AI and the accumulating evidence about its potential and impact in healthcare, obstacles to AI adoption persist, including regulatory issues, the digital divide, economic constraints, literacy levels and cultural resistance from users and administrative bodies. Furthermore, there is a lack of sufficient studies assessing medical doctors’ perspectives on this issue [41, 56, 57]. To address this gap, our nationwide survey aims to assess the potential and impact of AI in healthcare from the viewpoint of physicians with different specialties, experience, and working contexts in Portugal. Understanding these perceptions will contribute to promoting the inclusive adoption of AI in healthcare.

## Methods

### Study design, study population and questionnaire

We designed an observational, descriptive, cross-sectional study with a quantitative approach. In order to assess the perceptions of Portuguese physicians regarding the use of AI in healthcare we developed an online survey using the *SurveyMonkey* platform [58–60] entitled *“Artificial Intelligence in healthcare provision and the perspective of Portuguese physicians"*. This survey was composed of seven broad questions divided into "sub-questions", with response options in semantic differential—ranging from "1"(“strongly disagree") to "6" ("strongly agree"). Survey questions addressed the following aspects: extraction and processing of health data via AI; delegation of clinical procedures to AI tools; perception of the impact of AI in clinical practice; perceived advantages of using AI in clinical practice; perceived disadvantages of using AI in clinical practice and predisposition to adopt AI in their own professional activity. The survey also included 7 questions to assess the following dimensions of the respondents: demographic characterization (gender and age); professional characterization (medical specialty, years of experience and place of clinical practice); and digital use and proficiency characterization (use of ICT and self-perception of AI knowledge and digital technology command).

Our survey was pre-tested in a convenience sample of 11 doctors from different specialties (Pediatrics, Public Health, Nephrology, General Practice, Rheumatology and Oncology). As a result of this test, changes were introduced to the clarity of definitions, expressions and concepts included in the survey questions.

Link to the survey was sent via email to every licensed physician in Portugal via the Portuguese Medical Association, which reviewed the study protocol and survey and accepted to collaborate in its dissemination. Respondents were informed about the context and objectives of the study and were asked to voluntarily consent to participation (only by expressly selecting the consent option were respondents allowed access to survey questions). Respondents were also informed that the study was anonymous and that study results could be used for scientific publication purposes only.

Research ethics principles and legally applicable requirements were fully complied with.

### Data processing and statistical analysis

Considering that there are approximately 54,450 physicians officially licensed in Portugal, 656 valid answers would be required for a confidence level of 99% (p< 0.01), with a margin of error of 5% [61]. We obtained 1013 valid, fully answered questionnaires during the data collection period between September 18th and October 4th, 2019.

Results were transferred to IBM® SPSS® software (version 28) for data processing and statistical analysis. Univariate analysis consisting in the descriptive statistics of the sample and the frequency analysis of each variable was performed. In addition, score variables were built to facilitate bivariate statistical analysis (correlation tests) involving questions that contained semantic differentials (Questions 2, 3, 5, 6 and 7). To this end, responses "1", "2" and "3" were grouped into a larger category ("disagree"), and responses "4", "5" and "6" into a different larger category ("agree"). Each score was calculated using the arithmetic mean of the answers to the "sub-questions" included in each question, except for Question 4 (due to the lack of a unifying meaning among of the respective sub-questions, resulting in a low Internal Consistency). Each score was evaluated for Internal Consistency, using Cronbach’s alpha, revealing high consistency (Question 2 α = 0,975; Q3 α = 0,896; Q5 α = 0,934; Q6 α = 0,889; and Q7 α = 0,934 –S5 Table) [62]. Score variables were created for questions 13, with responses "never", "monthly", and "weekly" grouped into the category "low use," and responses "daily" into "high use." Additionally, for question 14, responses "1," "2," and "3" were grouped into "disagree," and responses "4," "5," and "6" into "agree".

In order to further facilitate the bivariate analysis, two dichotomous variables were built for Question 12 corresponding to place of clinical practice: Public (including National Health Service (NHS) Primary Care and NHS Hospital) versus Private (including Private Healthcare, Private Hospital and Private Offices/Clinics); and Primary Health Care (including Primary Health Care of the Portuguese NHS and Primary Health Care in a private unit) versus Hospitals (including Portuguese NHS Hospital, Private Hospital and Hospital of the social sector). A third category labelled "Other" (including Hospitals of the social sector, Insurance Companies/Subsystems, and private practices/clinics) was created to include places of professional practice which did not belong elsewhere and were also residual considering the total sample.

Bivariate analysis was conducted by assessing the statistical association between the variables under study through statistical tests. Non-parametric tests were used due to the non-normal distributions of the sample (confirmed by the Kolmogorov-Smirnov test). According to the characteristics of each variable, the non-parametric tests performed included: Spearman (correlation test) and Mann-Whitney U. Positive correlation between variables was considered when Sig (*p*-value) was smaller than 0.05 (for 95% confidence level).

## Results

### Demographic characterization of the study population

Demographic characteristics of the surveyed population are presented in Table 1. Survey respondents were mostly women (gender ratio 55,0: 45,0; female: male; n = 557 and n = 456, respectively) with a mean age of 46 years old (46,14 ±15,69). Most respondents were between 30 and 39 years old (25,6%), while only 6,5% of respondents were 70 or more years old (Table 1).

**Table 1:** Demographic and professional characteristics of respondents.

Characteristics	Category	Mean ± DP	
**Age**		46,14 ± 15,69	
**Age intervals** **N = 999**		**n**	**%**
	20–29 years old	183	18,3
	30–39 years old	257	25,6
	40–49 years old	134	13,4
	50–59 years old	153	15,3
	60–69 years old	209	20,9
	70 years old or older	63	6,5
**Gender** **N = 1013**	Masculine	456	45
	Feminine	557	55
**Setting of professional practice**	Hospital of the NHS	545	53,8
	Primary healthcare of the NHS	278	27,4
	Medical clinic/office of the private sector	265	26,2
	Hospital of the private sector	221	21,8
	Primary healthcare of the private sector	51	5
	Hospital of the social sector	25	2,5
	Insurance or health subsystem	24	2,4
	NHS sector	779	76,9
	Private sector	431	42,5
	Primary health Care	306	30,2
	Hospital Care	660	65,2
**Professional experience N = 981**	< 1 year	246	25,1
	1–9 years	215	21,9
	10–19 years	122	12,4
	20–29 years	176	18
	30–39 years	166	16,9
	> 40 years	56	5,7
**Medical specialty N = 1013**	General and Family Medicine	271	26,8
	Internal Medicine	70	6,9
	Pediatric Medicine	50	4,9
	Anesthesiology	45	4,4
	Medical Internship (Common year–no specialty)	40	3,9
	General Surgery	37	3,7
	Public Health	31	3,1
	Radiology	32	3,2
	Gynecology/Obstetrics	28	2,8
	Psychiatry	26	2,6
	Ophthalmology	24	2,4
	Clinical Pathology	20	2
	Orthopedics	20	2
	Occupational Medicine	19	1,9
	Rheumatology	19	1,9
	Physical and Rehabilitation Medicine	18	1,8
	Intensive Care Medicine	17	1,7
	Dermatology and Venereology	15	1,5
	Medical Oncology	16	1,6
	Psychiatry (Childhood and Adolescence)	13	1,3
	Cardiology	12	1,2
	Neurology	12	1,2
	Anatomic Pathology	11	1,1
	Immunology/Allergy	11	1,1
	Nephrology	11	1,1
	Otorhinolaryngology	11	1,1
	Neurosurgery	10	1
	Other	124	12,4

Descriptive statistics of the study population including age, gender, years of professional experience, setting of professional practice and medical specialty.

### Professional characterization of the study population

Physicians from all medical specialties were surveyed. The most represented specialty was General and Family Medicine (26,8%), followed by Internal Medicine (6,9%) and Pediatric Medicine (4,9%). For the sake of clarity and data treatment purposes, less represented medical specialties (< 1%) were grouped and classified as “Other” (Table 1).

In terms of professional experience as a medical specialist, most respondents had less than one year of experience (25,1%), followed by those with one to nine years’ experience (21,9%) (Table 1).

Regarding the setting of professional practice, most respondents worked in the Portuguese NHS (76,9%), either in a public hospital (53,8%), or on public primary healthcare (27,4%) (Table 1). 26,2% of respondents worked on a medical clinic/office of the private sector and 21,8% worked in a hospital of the private sector (Table 1). As medicine in Portugal is not mandatorily practiced in a regimen of exclusivity, respondents could select more than one setting of practice.

### Characterization of the study population in terms of use and command of Information and Communication Technologies (ICT)

**Fig 1:**
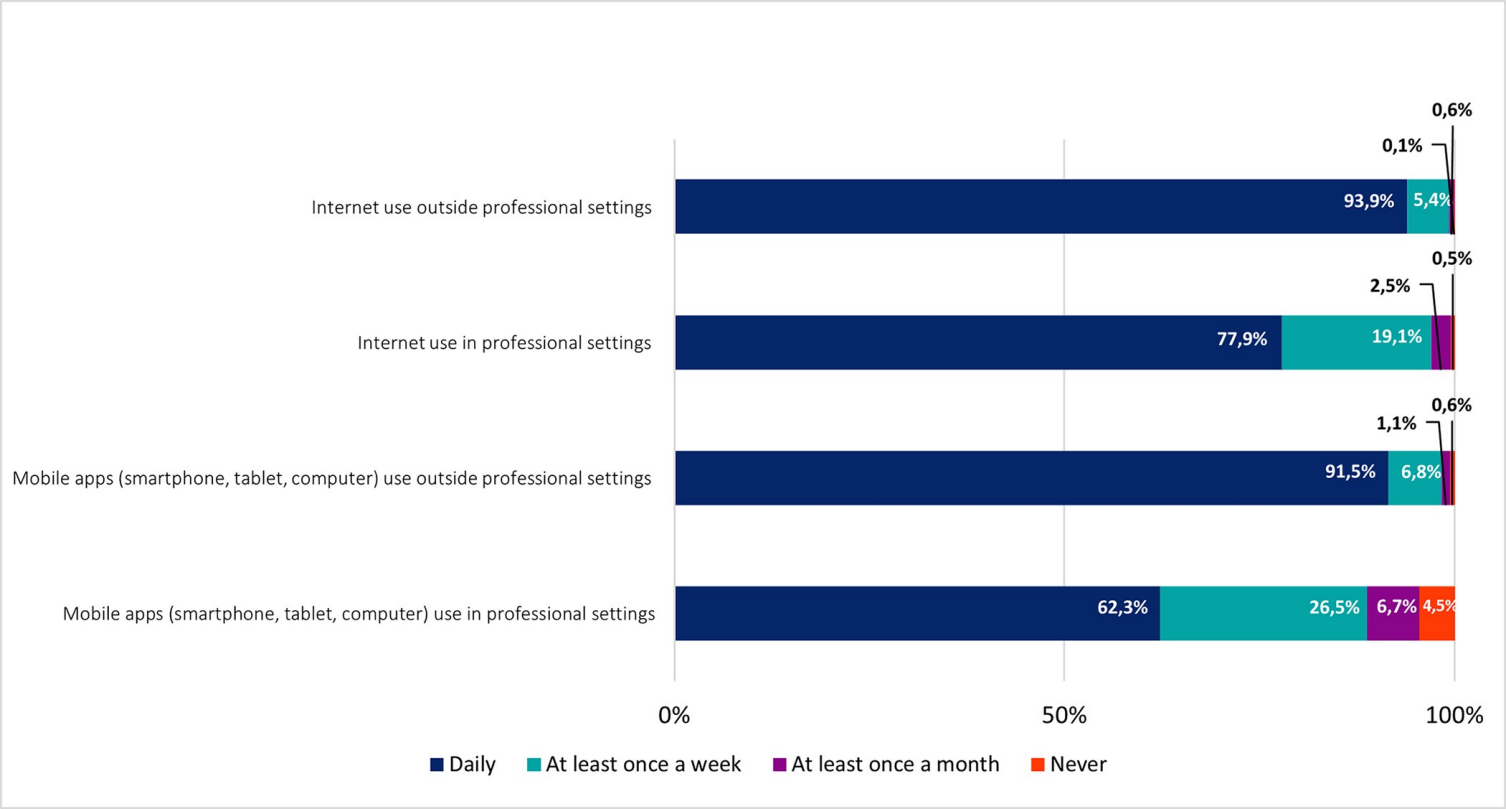
Use of information and communication technologies (ICT). Frequencies corresponding to periodicity of use of Information and Communication Technologies (ICT). Results correspond to answers to the question “How often do you perform each of the following activities?”.

Most respondents mentioned the daily use of the internet and mobile apps in non-professional settings (93,9% and 91,5%, respectively) (Fig 1). Although in smaller frequencies, most medical doctors in Portugal also referred the daily use of the internet and mobile apps in professional settings (77,9% and 62,3% of daily use, respectively). Nonetheless, most respondents mentioned using the internet (97%) and mobile apps (88,8%) in professional settings, either weekly or daily. Taken together our results show that medical doctors in Portugal are frequent users of ICT.

**Fig 2:**
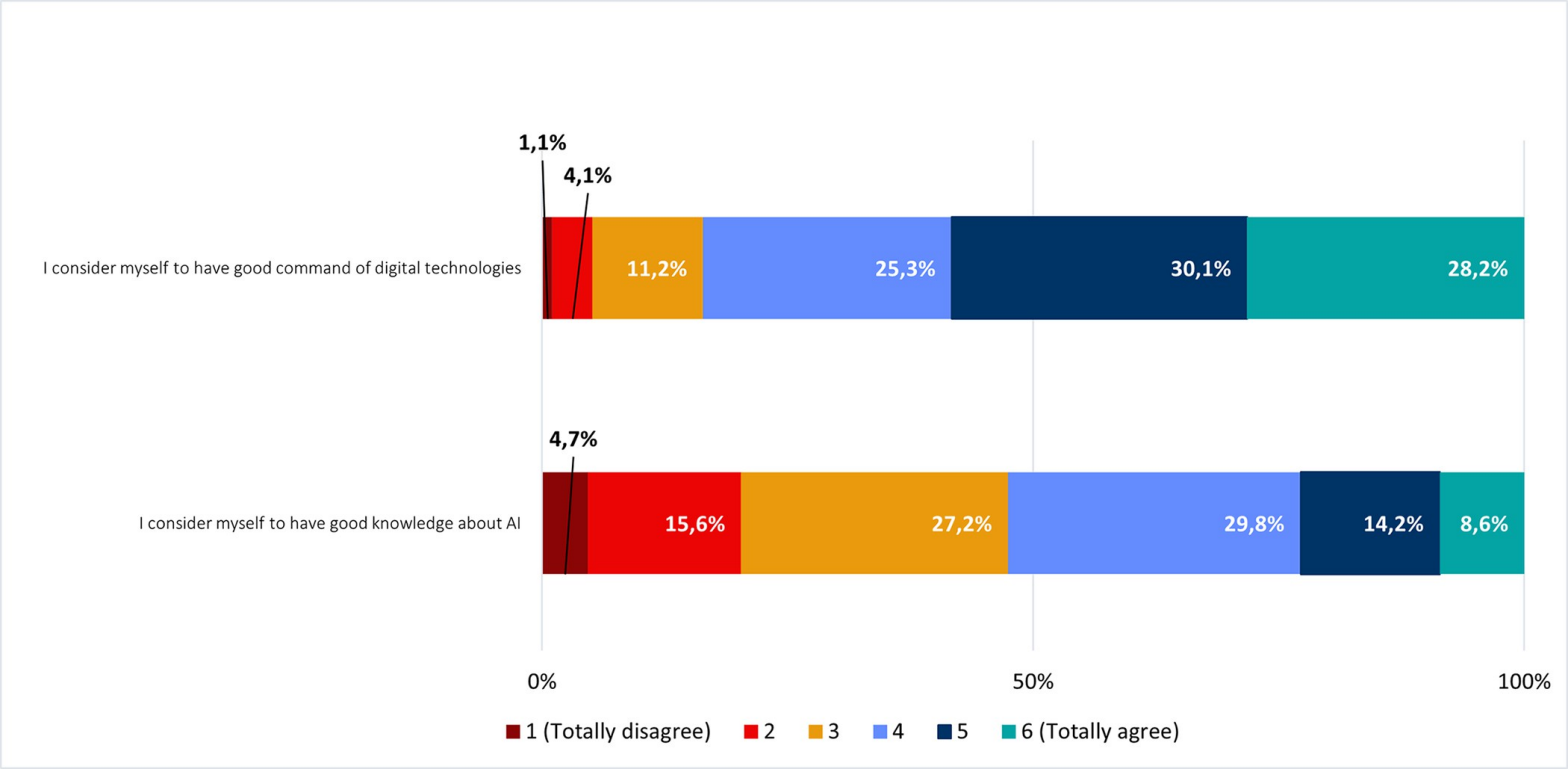
Self-perceived command of digital technologies and knowledge about AI. Frequencies corresponding to self-perceived command of digital technologies and knowledge about AI. Results correspond to agreement with statements on the left column, according to a Likert scale.

With the purpose of evaluating the self-perceived command of digital technologies and knowledge about AI, we questioned respondents to estimate their literacy levels according to a 6-level Likert scale [63, 64] (Fig 2). Most respondents (83,6%) considered to have good command of digital technologies (levels 4, 5 and 6 combined). In contrast, most respondents estimated their levels of knowledge about AI as being intermediate (29,8% for level 4 and 27,2% for level 3, resulting in 57% when combined).

### Perceptions regarding the impact of AI on healthcare

**Fig 3:**
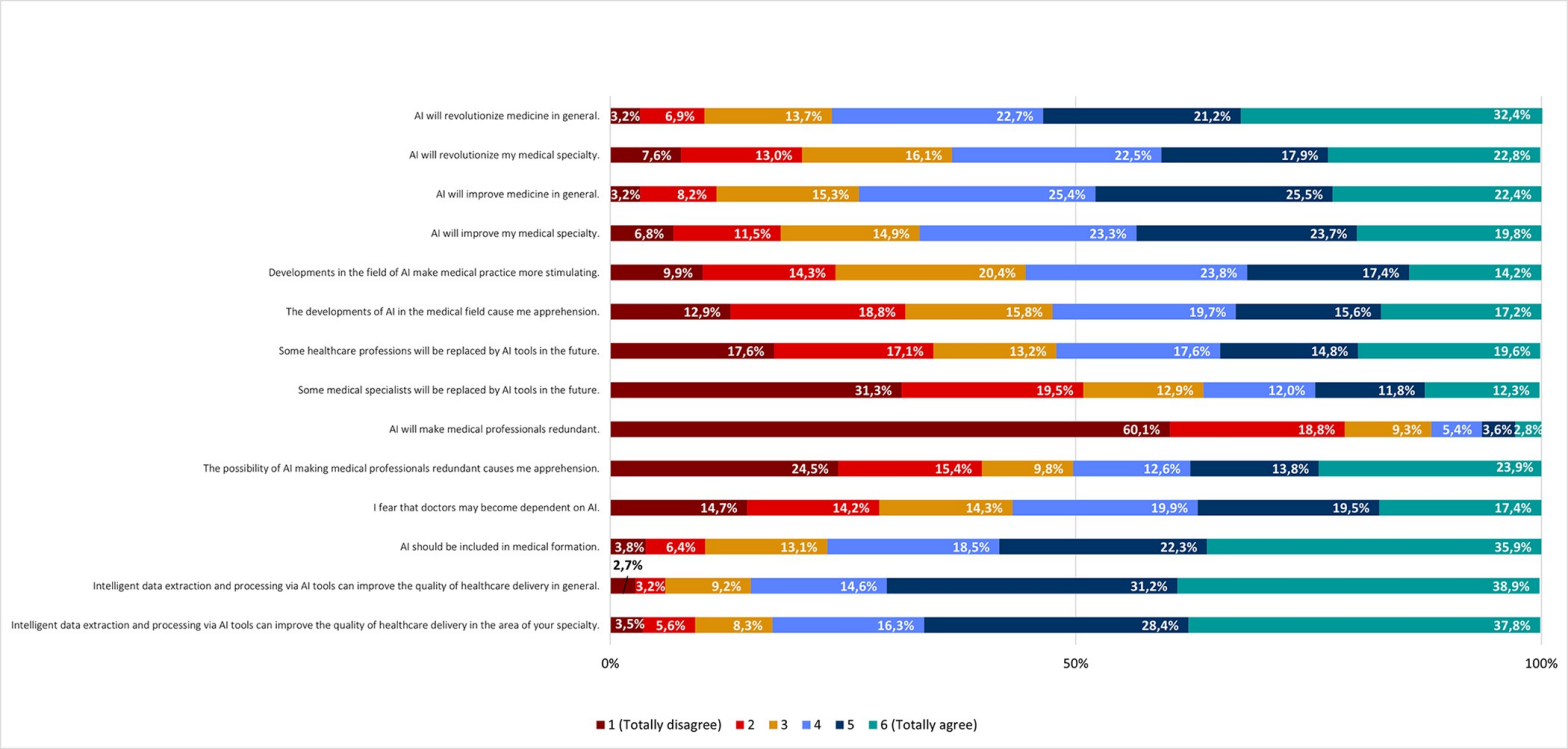
Impact and potential of the use of AI in healthcare delivery. Frequencies corresponding to impact and potential of the use of AI in healthcare delivery. Results correspond to agreement with statements on the left column, according to a Likert scale.

To evaluate the perceptions of medical doctors in Portugal regarding the impact of AI in healthcare, we first asked respondents to indicate their agreement or disagreement (according to a 6-level Likert scale) with a series of related statements. Most respondents agreed that AI will have an impact in medicine in general. In particular, 76,3% agreed (32,4% totally agree; only 3,2% totally disagree) that AI will revolutionize medicine and 73,3% agreed (22,4% totally agree; only 3,2% totally disagree) that it will improve it (Fig 3). Accordingly, most respondents also agreed that AI should be included in medical formation (76,7% as a combination of levels 4,5 and 6; 35,9% totally agree; 3,8% totally disagree). Regarding the impact of AI on their own medical specialties, respondents were slightly less assertive. Nonetheless, 63,2% still agreed that AI will revolutionize their specialty and 66,8% agreed that it will improve it. Furthermore, 55,4% agreed that developments in AI will make medical practice more stimulating (Fig 3).

Despite the assessment that AI will have a significant impact in the medical profession, 88,2% of respondents disagreed (60,1% totally disagree) with the general statement that AI will make medical doctors redundant. However, disagreement was reduced to 63,7% regarding whether medical specialists will be replaced by AI in the future and, notably, 52% of respondents agreed that some healthcare professions will be replaced by AI in the future. Furthermore, approximately half of those inquired (50,3%) agreed that the possibility of medical doctors becoming redundant due to AI causes apprehension as do the developments of AI in the medical field (52,5%). Accordingly, 56,8% of respondents fear that medical doctors may become dependent on AI (Fig 3).

Specifically, regarding the potential of AI-mediated health data processing, 84,7% of respondents agreed with a resulting improvement of the quality of healthcare delivery in general (38,9% totally agree), and 82,5% agreed (37,8% totally agree) with an improvement in their specific specialty (Fig 3).

### Perceptions regarding the use of AI in healthcare

This study also aimed to assess physicians’ perceptions regarding the use of AI in healthcare according to four different dimensions: delegation of tasks, specific advantages, specific disadvantages, and predisposition to adopt AI use.

#### I. Task delegation

**Fig 4:**
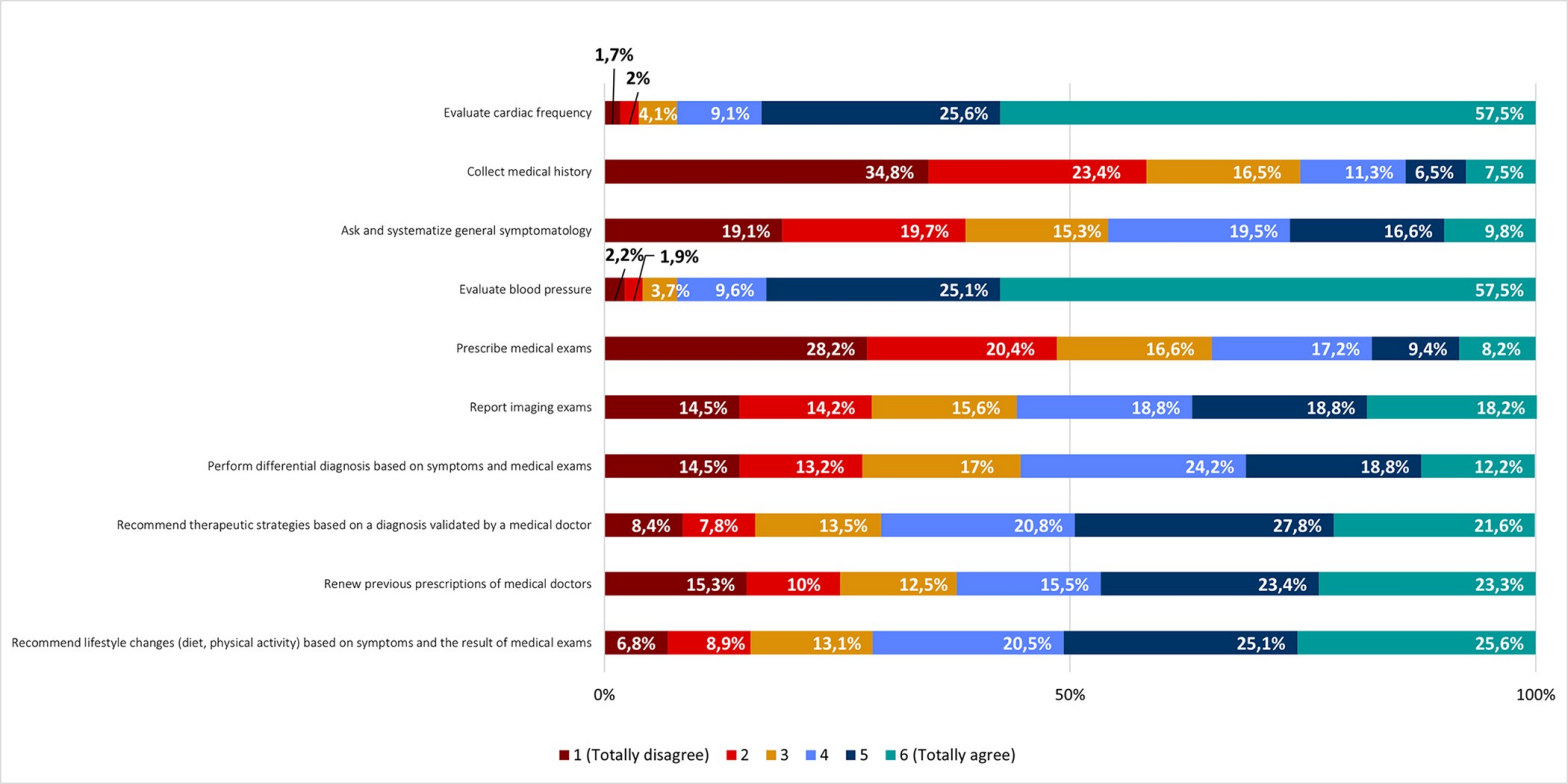
Task delegation on AI tools. Frequencies corresponding to task delegation on Artificial Intelligence tools. Results correspond to agreement with statements on the left column (by responding to the following question: “*Among the following procedures*, *please indicate the degree of agreement that it can be delegated to an AI tool”*), according to a Likert scale.

Respondents indicated that they agree to delegate various tasks on AI, including evaluating cardiac frequency (92,2%; 57,5% totally agree), and blood pressure (92,2%; 57,5% totally agree); recommending lifestyle changes based on symptoms and the result of medical exams (71,2%); recommending therapeutic strategies based on a diagnosis validated by a medical doctor (70,2%); renewing previous prescriptions of medical doctors (62,2%); reporting imaging exams (55,8%); and performing differential diagnosis based on symptoms and medical exams (55,2%) (Fig 4).

On the contrary, most respondents disagreed about delegating other tasks on AI, in particular, obtaining medical history (74,7%; 34,8% totally disagree); prescribing medical exams (65,2%; 28,2% totally disagree); and asking and systematizing general symptomatology (54,1%) (Fig 4).

#### II. Specific advantages

**Fig 5:**
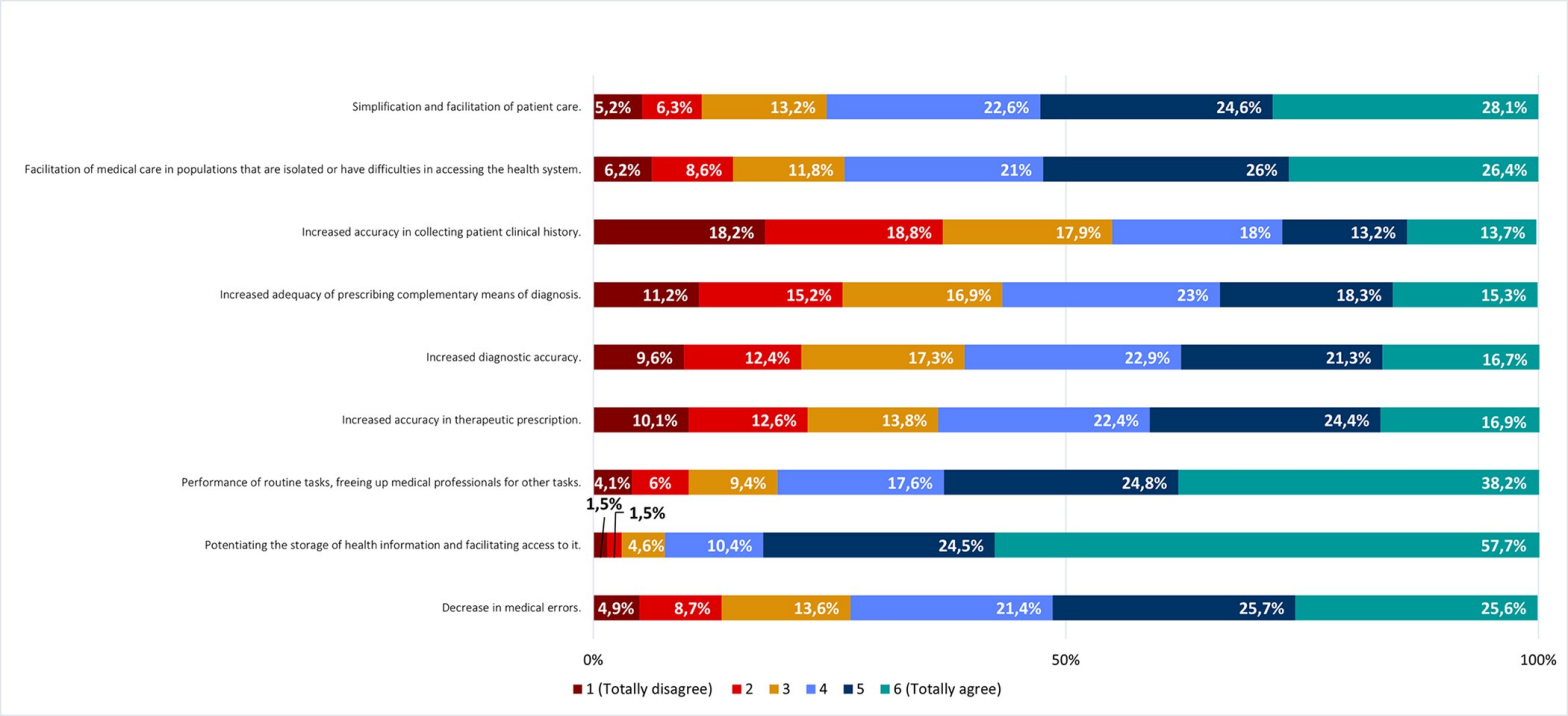
Specific advantages of using AI in healthcare. Frequencies corresponding to agreement/disagreement with specific advantages of using Artificial Intelligence tools in healthcare. Results correspond to agreement with statements on the left column (by responding to the following question: *“Please indicate your degree of agreement with the following possible advantages of using AI tools in healthcare"*), according to a Likert scale.

Respondents agreed with different advantages of using AI, such as potentiating the storage of health information and facilitating access to it (92,6%); fulfilling routine tasks, while freeing medical professionals for other tasks (80,6%); simplifying and streamlining patient care (75,3%); facilitating access to healthcare for isolated populations or others (73,4%); reducing medical errors (72,7%); increasing accuracy in therapeutic prescriptions (63,7%), diagnosis (60,9%), and the adequacy of the prescription of complementary diagnostic procedures (56,6%) (Fig 5).

On the contrary, in line with their disagreement in delegating the collection of medical history on AI (Fig 4), respondents disagreed that AI has the advantage of increasing the accuracy in collecting patient clinical history (55,2% disagree; 18,2% totally disagree) (Fig 5).

#### III. Specific disadvantages

**Fig 6:**
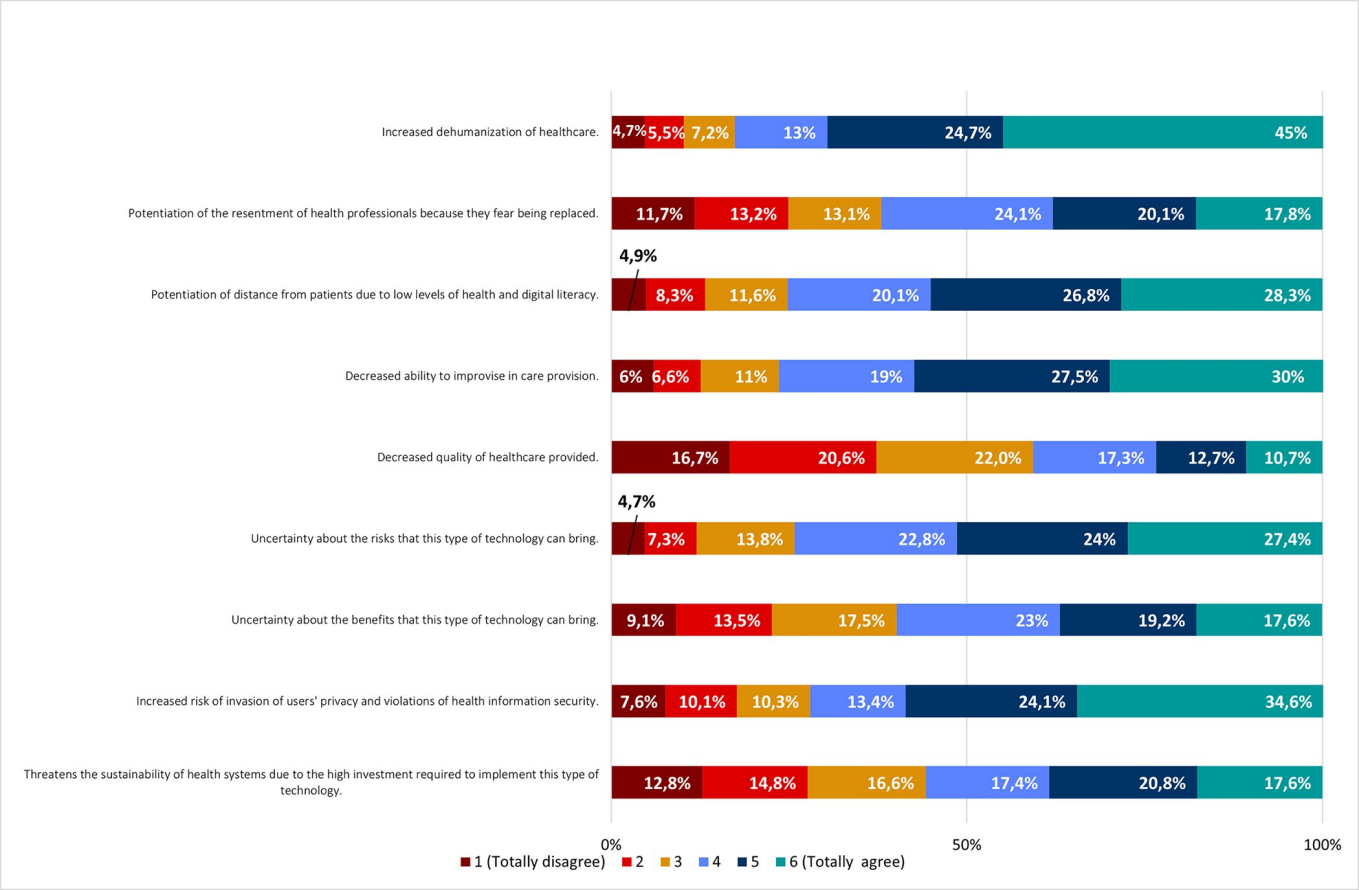
Specific disadvantages of using AI in healthcare. Frequencies corresponding to agreement/disagreement with specific disadvantages of using Artificial Intelligence tools in healthcare. Results correspond to agreement with statements on the left column (by responding to the following question: *“Please indicate your degree of agreement with the following possible disadvantages of using AI tools in healthcare"*), according to a Likert scale.

Regarding disadvantages of AI use, our results revealed a general agreement among respondents. Increased dehumanization of health care (82,7%); decreased ability to improvise in care provision (76,5%); potentiation of distance from patients due to low levels of health and digital literacy (75,2%); uncertainty about the risks (74,2%); increased risk of invasion of users’ privacy and violations of health information security (72,1%); potentiation of resentment of health professionals due to fear of being replaced (62%); uncertainty about benefits (59,8%); and threat to the sustainability of health systems due to high investment required (55,8%), were agreed to as disadvantages of AI use in healthcare (Fig 6).

**Fig 7:**
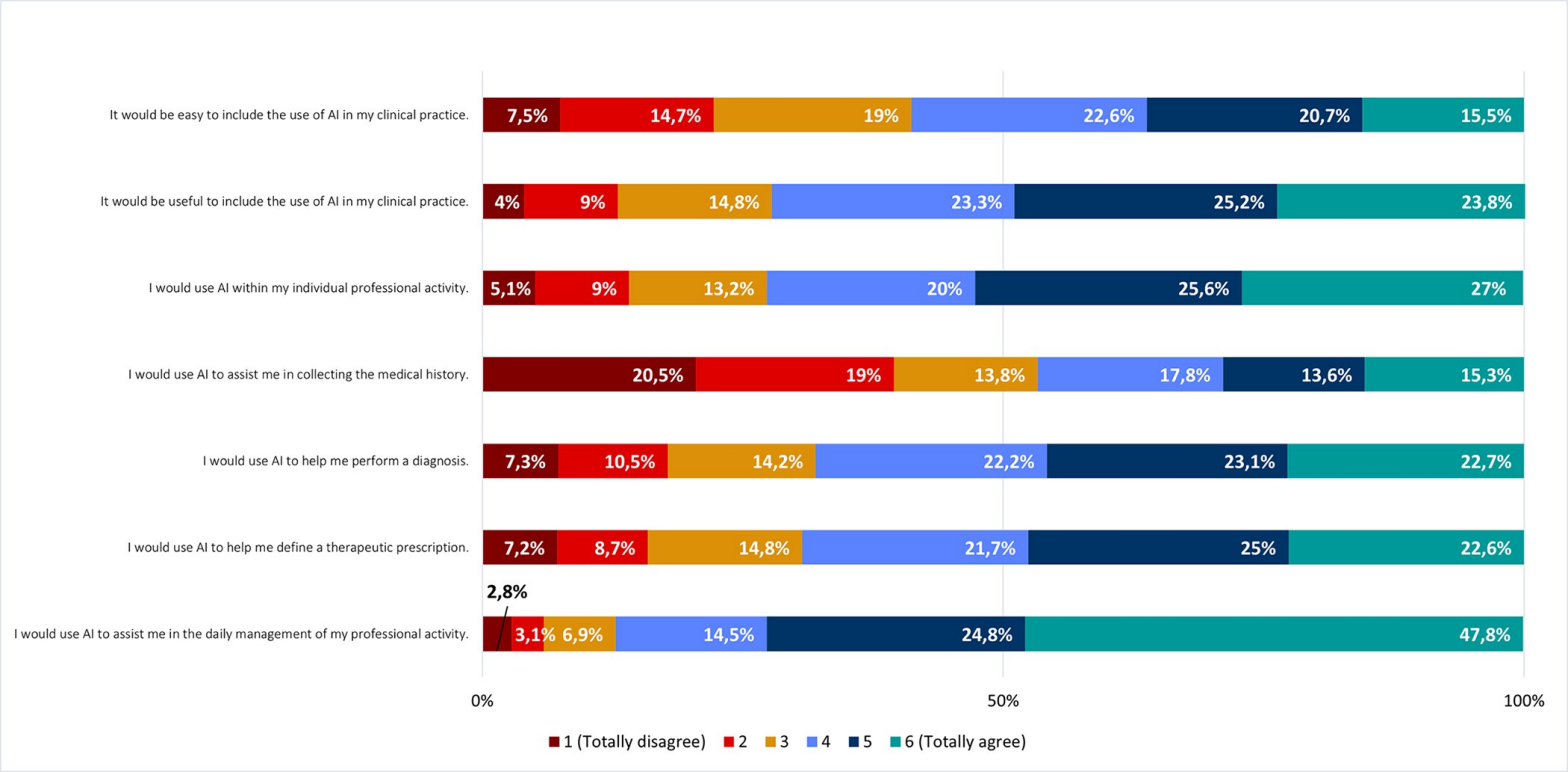
Predisposition to adopt AI in clinical practice. Frequencies corresponding to agreement/disagreement with predisposition to adopt AI in clinical practice. Results correspond to agreement with statements on the left column (by responding to the following question: *“Please indicate your agreement with the following statements regarding the use of AI in your clinical practice”*), according to a Likert scale.

*I*. *Predisposition to use AI in clinical practice*. Considering the predisposition of using AI in their own clinical practice, respondents agreed that it would be useful (72,3%; 23,8% totally agree) and easy (58,8%; 15.5% totally agree) (Fig 7). Accordingly, most respondents admitted they would use AI in their professional activity (72,6%; 27% totally agree). Specifically, respondents admitted they would use AI in the daily management of their professional practice, for example writing in the electronic health record, schedule, and book appointments (87,1%; 47,8% totally agree). Most respondents also admitted they would use AI to assist them in defining therapeutic prescriptions (69,3%; 22,6% totally agree) and making diagnoses (68%; 22,7% totally agree). In accordance with responses to previous questions (Figs 4 and 5), respondents were divided about using AI to assist them in collecting patient’s medical history (53,3%; 20,5% totally disagree) (Fig 7).

### Bivariate analysis: Correlation and association between different AI perceptions and study population characteristics

Through bivariate analysis of our results, we found a positive and statistically significant association between the general (professional and non-professional) use of ICT and the perception that application of AI in health data extraction and processing improves healthcare quality (ρ = 0.252; *p <* 0.001). Respondents with higher digital and AI command also agreed more with this perception (ρ = 0.158; *p <*0.001) (S1 Table). We also found a significant association between gender and the perception that application of AI in health data extraction and processing improves healthcare quality (*p<*0.001) (S6 Table), with men tending to agree more (88,7%) compared to women (83,7%) (S2 Table). Contrarily, no association was found between this AI perception indicator and the respondent’s place of clinical practice (S6 Table). Regarding the delegation of clinical procedures to AI tools, the higher the respondent’s general (professional and non-professional) ICT use (ρ = 0.170; *p <*0.001) and AI and digital command (ρ = 0.096; *p <*0.001), the higher the agreement with delegation to AI (S1 Table). On the other hand, we also found that men agree more (76,0%) than women (59,9%) (S2 Table) with the delegation of clinical procedures to AI tools (*p* < 0.001) (S6 Table).

**Fig 8:**
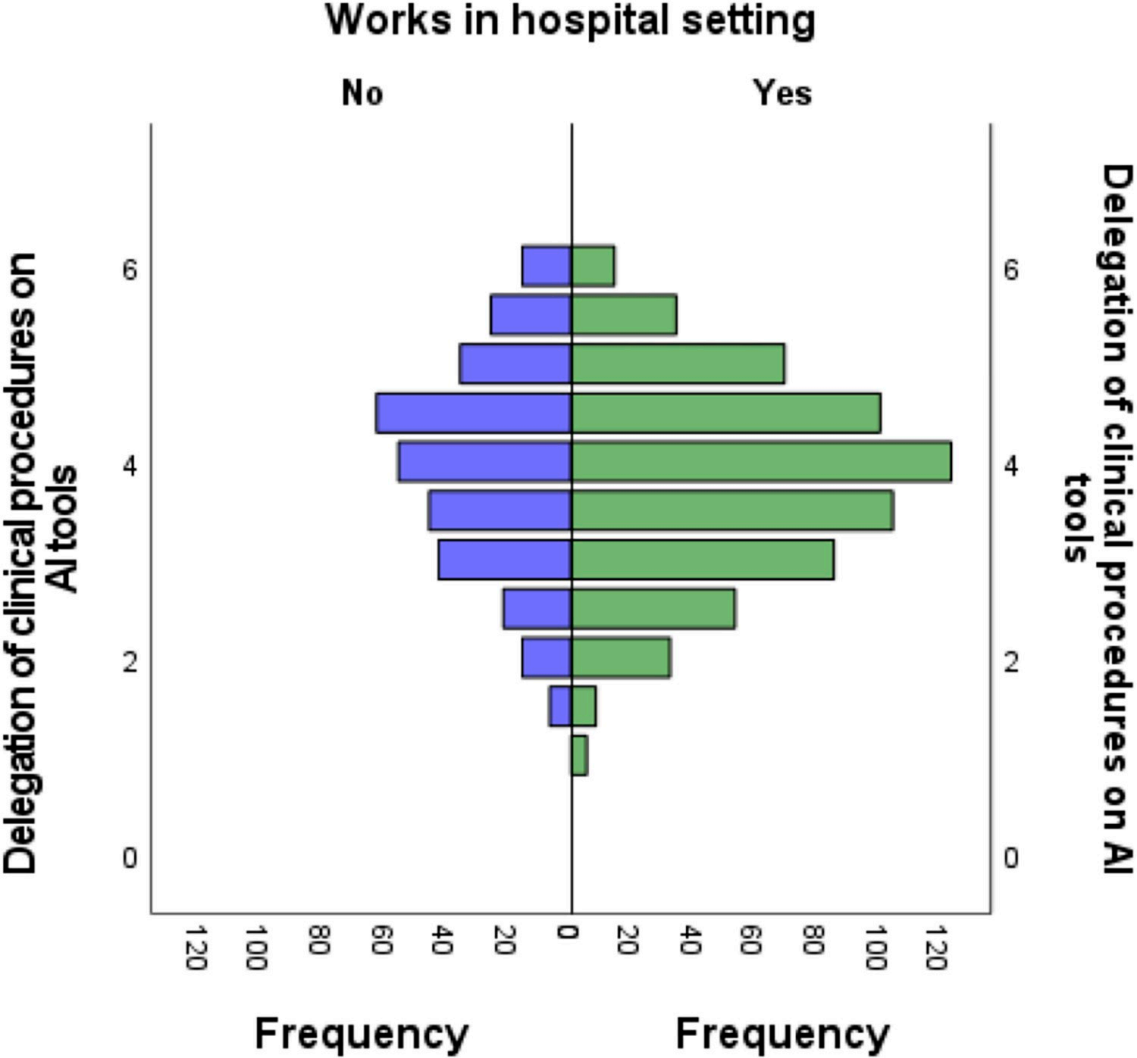
Agreement to delegate clinical procedures on AI tools as expressed by medical doctors who work in hospital settings. Association between the score corresponding to question 3 *“Delegation of clinical procedures on AI tools”* and results of respondents who work in hospital settings (*p*<0.05). Independent samples of Mann-Whitney U test.

We also found a statistically significant association (ρ = 0.119; *p*< 0.001) between age and delegation of clinical procedures to AI tools, indicating that the older the respondent the higher the expressed agreement with delegation to AI (S1 Table). In accordance with this observation, the respondents with more years of professional experience expressed higher agreement with delegation of clinical procedures to AI (ρ = 0.113; *p*< 0.001) (S1 Table). Furthermore, respondents who work in a hospital environment expressed higher agreement with delegation of clinical procedures to AI (65,45%) (Fig 8) (*p* < 0.05) (S6 Table). In contrast, no statistically significant relationship was observed between this AI perception indicator and respondents who work in primary care, in the public sector or private sector (S6 Table).

Regarding the perceived advantages of AI in healthcare, we found a statistically significant association between this indicator and gender, with men perceiving more advantages than women (85,1% and 73,1%, respectively) and, conversely, women perceived more disadvantages than men (85,6% and 68,1%) (S2 and S6 Tables).

However, ICT use (ρ = 0.204; *p*< 0.001) and digital and AI command (ρ = 0.141; *p*< 0.001), have revealed statistical significance, both with positive correlation coefficients. Consistently, respondents who expressed higher agreement with the delegation of clinical procedures to AI, perceived more advantages (ρ = 0.771; *p* < 0.001) and less disadvantages (ρ = -0.276; *p* < 0.001) of AI use in healthcare (S1 Table). Furthermore, the higher the ICT use (ρ = -0.202; *p* < 0.001) and digital and AI command (ρ = -0.101; *p* < 0.001) of respondents, the lower their perceived disadvantages in AI use in healthcare (S1 Table).

Additionally, higher ICT use, and digital and AI command corresponded to higher predisposition to adopt AI tools in clinical practice (ρ = 0.263; *p*< 0.001 and ρ = 0.139; *p*< 0.001, respectively) (S1 Table). As observed for other AI use in healthcare indicators, we also found a statistically significant association between gender and predisposition to adopt AI in clinical practice (*p* < 0.001) (S6 Table), with men agreeing the most (81,8%) when compared to women (68,4%) (S2 Table). In our study, we did not find significant differences between medical specialties regarding the various AI perception indicators assessed. However, we did observe a statistically significant result (p<0,033) for Radiologists regarding the "predisposition for using AI in clinical practice" indicator compared to other medical specialties (S3 Table). This suggests that radiologists might have a higher inclination to utilize AI tools in their clinical practice compared to other medical specialties.

Finally, respondents who affirmed higher predisposition for AI adoption in their clinical practice also express higher agreement with delegation of clinical procedures to AI tools (ρ = 0.689; *p* < 0.001) and agree more that application of AI in health data extraction and processing improves healthcare quality (ρ = 0.661; *p* < 0.001). The respondents who perceived more advantages in AI use agree the most with adopting AI in their clinical practice (ρ = 0.819; *p* < 0.001). Conversely, those who perceived more disadvantages in AI use are those with less predisposition for AI adoption in their clinical practice, as these results reveal a statistically significant association with a negative correlation (ρ = -0.373; *p* < 0.001) (S1 Table).

## Discussion

This study aimed to assess the perception of Portuguese medical doctors regarding the potential and impact of AI in healthcare. Our survey reached all licensed medical doctors in Portugal, with a higher representation of General Practice and Family Medicine specialists (26.8%), which aligns with the national distribution [65].

Most respondents in our study reported being frequent digital users and having good knowledge of digital technologies. However, their knowledge of AI was generally rated as intermediate, possibly because AI is a relatively new field compared to other digital technologies. This finding is consistent with previous research in healthcare [63, 66–70].

In our study, respondents considered that most tasks can be delegated to AI tools, particularly medical doctors working in hospital settings. This finding might reveal lower risk aversion and/or optimism toward the potential of technology, perhaps due to the fact that medical doctors working in hospitals have more contact with technology [71].

Obtaining medical history and prescribing medical exams were the tasks that most respondents showed less agreement to delegate. Consistently, most respondents did not consider that AI has the advantage of increasing the accuracy in performing these tasks. In the future it would be interesting to continue to explore why it is that obtaining medical history is perceived by medical doctors as a less-delegable task. It could be due to its inherent complexity, its variability, or the tendency to result from a direct communication between the doctor and the patient. It has long been established that good communication and empathy skills are fundamental to strengthen the (human) doctor-patient relationship and improve healthcare quality [72–74]. Furthermore, different evidence has highlighted the collection of medical history as a limitation of digital health [75, 76]. In contrast, AI is currently used to deliver personalized patient care, which is usually perceived as a synonym of higher accuracy [23, 77, 78]. Other studies have shown that the use of AI tools to take medical history has advantages, for example, the interaction is not time-constrained which will not limit patient response times to seconds, the fact that computers do not use heuristics and the fact that these systems may increase the quality of care by supporting the optimization of staff assignments [79, 80].

Most respondents did not consider that prescribing complementary diagnostic tests can be delegated to AI tools. Nonetheless, using these tools to adjust complementary diagnostic prescription is perceived as an advantage. These results can demonstrate that medical doctors view AI essentially as a support instrument (a very valuable assistant or reviewer) but not as a substitute, which highlights a mixture of trust and skepticism [81]. Other results from this study are consistent with this hypothesis: simpler routine clinical activities such as checking blood pressure and heart rate obtained greater agreement for delegation on AI tools. Moreover, the most perceived advantage of AI was the increased storage of health information and the facilitation of health data access, which is compatible with the use of AI to assist the daily management of professional activity and also with the previously documented predisposition for delegating on AI the systematization of the patient’s general symptomatology [41].

While most respondents believe that their specific occupation will not be replaced by AI in the future and their profession will not become redundant, they also express concerns about the potential resentment caused by AI implementation among medical doctors, fearing their own replacement as a disadvantage of using AI in clinical practice. Additionally, many respondents acknowledge the possibility of AI replacing other healthcare professionals, aligning with existing evidence that foresees the impact of AI on certain healthcare professions [24, 48, 82–84]. For example, in the field of Radiology, evidence shows that using AI has broader implications outside the traditional activities of lesion detection and characterization [85]. Nonetheless, in alignment with similar studies that assessed physician´s perspectives on AI utilization, our research revealed that physicians demonstrated a positive attitude about integrating AI into their practice. They did not primarily express concern about being replaced by AI, and believe AI training and education should be enhanced [86–89].

However, variations within the medical profession exist. For example, other studies indicate that surgeons are less worried about job replacement compared to other specialties due to the intricate surgical procedures and direct interaction with patients that their role entails [42]. Despite this fact, our analysis did not reveal significant differences between medical specialties. However, it is worth noting that in our study radiologists reported a higher inclination to adopt AI tools in their clinical practice compared to other medical specialties. While not present for all indicators assessed, this difference observed for radiologists aligns with the more established use of AI in this medical specialty compared to others [90–93].

Our study showed that most physicians believe that AI could reduce medical errors. AI technology is capable of highlighting hidden health information in large databases in order to aid clinical decision-making, which can help reduce misdiagnoses that are inevitable to human practice [5, 14, 94, 95]. Furthermore, AI use in clinical practice can significantly relieve the pressure of routine work [41], freeing up more time for patient care and quality improvement [40, 82, 96]. Nonetheless, the fact that many participants in our study believe that AI can dehumanize medical care highlights the need to implement mechanisms that ensure automation-driven efficiency gains in healthcare do not equate to devaluing human interaction [24]. A balanced and complementary human-machine interaction can foster dialogue and proximity between doctors and patients, especially when clinicians are released from routine work and are better equipped with knowledge resulting from the analysis of large amounts of data [97]. Big data, which must be clean and readily available for analysis by AI systems. Additionally, bias detection, correction, and data contextualization are crucial steps that must be carried out [98].

Ideally, achieving this balanced human-machine interaction would require a collaborative effort, involving healthcare professionals, technical experts, policy makers, and patients to establish guidelines, standards, and ethical considerations that ensure the effective and responsible integration of AI in healthcare.

Another key finding from our study is that medical doctors practicing in Portugal believe that AI can facilitate healthcare access for isolated populations. Importantly, providing care to isolated populations and improving mobile connectivity through technological devices, can enable clinicians and public health researchers to better understand variability between individuals and populations, providing more individualized care and planning more appropriate preventive therapeutic measures [23, 28, 78, 99, 100]. Concomitantly, patients armed with their own health data (combined with information from central databases) can better communicate with health professionals and feel more empowered to access healthcare [101]. On the flipside, risks of privacy invasion and security breaches of health information can diminish trust in digital health and put patients of technology adoption [22]. Our results show that privacy and security risks are perceived by medical doctors in Portugal as a downside of AI. Consistent with other studies, the successful integration of AI in medicine relies on the establishment of robust data privacy and security safeguards [19, 77, 98, 102, 103]. This imperative extends to the perspectives of African radiographers who also express concerns about job security and data protection, which emphasizes the critical need to address these and other issues for the successful implementation of advanced medical imaging technologies on a global scale [104, 105].

Globally, AI implementation in healthcare has been proposed to depend on success factors, including policy setting, technological implementation, and medical and economic impact measurement, each of which leading to specific recommendations, including risk-adjustment and “privacy by design” policies as well as quantification of medical and economic impact [106].

In the EU, healthcare is a priority sector for AI implementation, and countries proposed regulatory frameworks for health data management. However, specific policies vary, and some are still being defined [107].

Furthermore, AI adoption in healthcare remains limited to certain areas, partly due to insufficient trust in AI-driven decision support, which differ among countries [108].

Addressing country-specific and cultural concerns regarding AI use in the healthcare sector is crucial. Reported disparities in AI scientific output, collaboration, and adoption between larger and smaller EU countries have prompted experts to call for coordinated approaches at the European level [107].

A significant finding in our study was that some respondents perceived a disadvantage of AI in healthcare as the reduced ability to improvise, which is a skill that medical doctors develop through years of practice [109]. As AI implementation gathers pace, it is fundamental to understand its impact at this level. Representing improvisation (or intuition) in healthcare as an exclusively human quality calls for a careful reflection in light of the nature of the medical act in its different dimensions, including risk, uncertainty, and responsibility [110]. AI-based systems, with their learning and self-correction capabilities, have the potential to enhance accuracy in health outcomes by incorporating feedback and extracting information from a large user population for real-time risk warnings and diagnostic predictions [5, 41]. In parallel, the risk of automation bias is highly associated with the cases when clinicians become overly dependent of decision support systems, which can lead to de-skilling of human professionals [111]. Studies suggest the importance of maximizing the benefits of AI tools while minimizing over-reliance through the implementation of constant checking alerts that prevent unchecked decisions [111, 112].

Our results also show that, as expected, the more willing a medical doctor is to use AI in their clinical practice and the more predisposed they are to delegate clinical tasks to AI tools, the more advantages (and less disadvantages) they perceive in using AI in healthcare. This is coherent with a view of AI use in medicine built on a human-machine trust relationship, which respects ethical principles and potentiates AI adoption [113–117].

Furthermore, in our study the older and more experienced the respondents, the higher agreement to delegate clinical tasks on AI. This is an interesting finding. Further investigation should focus on the specific reasons for this result, but one can speculate that experience in clinical practice may lead to better prioritization, easier recognition of medical tasks that may be delegable on AI tools and a higher human focus on procedures that are considered not to be not delegable on AI. Also, some studies have shown that beliefs related with older adults being less compatible with digital health innovation than the younger [118]. Care must be exercised not to expand the same idea to professional adoption, which could disproportionately exclude more experienced professionals from AI use.

The advancement and implementation of AI technologies in medical imaging should be accompanied by adequate professional training. This applies not only to medical specialties like radiology, which lead in AI implementation due to their technologically enhanced nature, but also extends to other medical specialties [119]. As demonstrated in the literature, while there are numerous benefits of AI-enabled clinical workflows, such advancements can also bring about disruption of traditional roles and patient-centered care [24, 46, 120]. However, these challenges can be managed by promoting the education of the medical workforce [46, 50, 119]. Therefore, it is widely recognized that healthcare professionals need to improve their digital and AI literacy to effectively adopt AI in clinical practice [24, 46]. Our study underscores the perception that AI should be an integral part of medical training, emphasizing the need to educate all stakeholders about AI applications, limitations, and specific challenges [46, 50]. Universities and healthcare courses play a central role in facilitating this transition and promoting the inclusion of AI in medical education [53, 102]. Notably, AI integration in medical education is already evident in certain specialties like Radiology, owing to the extensive use of medical imaging and diagnostic possibilities [53, 102]. The results of our study align with other evidence indicating that medical doctors are receptive to learning more about AI and consider it important for their practice [45, 48, 49, 53–55, 121]. Considering the rapid proliferation of AI in healthcare, it is crucial to accelerate the inclusion of digital health disciplines in medical degrees to prepare future healthcare professionals for the challenges and opportunities presented by AI [122].

Our study has significant implications for research, policy, and practice. By surveying a large sample of medical doctors, we identified an overall optimism about the potential of AI and a significant predisposition for AI adoption, which highlights the need to further research the nature of the obstacles and resistance to AI use that still exist. Future policy should focus on maximizing the potential of AI use in clinical practice considering the views of health professionals, while urgently addressing identified challenges, in particular the existing gaps in medical curricula, which limit the use of state-of-the art AI applications in healthcare due to suboptimal digital health training.

### Limitations

Respondents were surveyed using a digital online questionnaire, which can limit the participation of some potential responders. Most responders had less than 10 years’ experience. Nonetheless, we believe that this reflects the national reality. Furthermore, our sample size takes into account the number of licensed physicians in Portugal, guaranteeing a statistical confidence level of 99% (with a margin of error of 5%) within the specific context of Portugal. Cultural and context specificities of clinical practice in Portugal should be considered. The fact that many respondents accumulate jobs between public, private or social work settings may limit conclusions about professional environments. Extrapolation of the results of this study to other realities should be carried out in a rigorous and controlled way. AI implementation is influenced by various factors that go beyond physician perceptions. These factors include patient literacy levels, systems interoperability, health reimbursement arrangements, legal and regulatory contexts, among others. Nonetheless, due to high levels of professional integration in the healthcare sector, at least within the European Union, our results should also be relevant for analysis in other contexts.

### Conclusions

This study concludes that medical doctors licensed to practice in Portugal are in agreement about the significant impact of AI on medicine, including their own specialty, and advocate for its inclusion in medical training. AI is seen as a valuable tool for healthcare professionals, offering benefits such as improved access to health data, increased accuracy, reduced errors, and relief of medical doctors from routine tasks, thereby enhancing the quality of healthcare. However, physicians reported resistance to utilizing AI for obtaining medical history and prescribing tests. Concerns were raised about potential dehumanization of healthcare, decreased ability to improvise when necessary, and risks to privacy and confidentiality. Interestingly, older and more experienced doctors displayed greater optimism and openness towards incorporating AI in medicine.

By conducting a nationwide survey of physicians´ perspectives, this study significantly contributes to facilitate the professional integration of AI in medical practice. The high professional integration observed in the healthcare sector, especially within the European Union (EU), makes our results relevant for broader geographical contexts and diverse healthcare systems. Furthermore, our findings emphasize the importance of expediting the optimization of AI use in healthcare and the integration of AI skills training into medical curricula.

## Supporting information

S1 Table Correlation (Spearman) between different AI perceptions and study population characteristics. Coefficients of correlation between AI perceptions (scores) and different study population characteristics. ns. = not significant; **Correlation is significant at the 0.01 level (2-tailed); * Correlation is significant at the 0.05 level (2-tailed); AI in DEP—Application of AI in health data extraction and processing (Question 2); Delegation on AI—Delegation of clinical procedures on AI tools (Question 3); Adv. of AI—Specific advantages of AI (Question 5); Disadv. of AI—Specific Disadvantages of using AI (Question 6); Pred. for using AI—Predisposition for using AI in clinical practice (Question 7); ICT use–use of information and communication technologies (Question 13); Com. of DT and AI—Self-perceived command of digital technologies and knowledge about AI (Question 14); YPE—Years of Professional Experience.
(DOCX)

S2 Table Indicators of AI perceptions (scores) according to gender. Description of score results according to gender (statistically significant associations).
(DOCX)

S3 Table Indicators of AI perceptions (scores) according to medical specialties (radiology). Description of score results according to radiology and other medical specialties (statistically significant associations).
(DOCX)

S4 Table Descriptive statistics of the indicators of AI perceptions (scores). Description of general statistics related with the scores and their respective questions.
(DOCX)

S5 Table Internal consistency of the questionnaire. Results of Cronbach´s Alpha test (questions 2, 3, 5, 6 and 7 of our survey) for measure of internal consistency (reliability test), and the respective coefficient´s level (Coefficient of Cronbach´s Alpha: more than 0.9—Excellent; 0.80–0.89 –Good; 0.70–0.79 –Acceptable; 0.6–0.69 –Questionable; 0.5–0.59 –Poor; less than 0.59 –Unacceptable). 1* Corrected total item correlation [62].
(DOCX)

S6 Table Associations between different AI perceptions and study population characteristics (Mann-Whitney U). U-test values for statistically significant associations between AI perceptions (scores) and different study population characteristics. ns. = not significant; ** Significant at the 0.01 level (2-tailed); * Significant at the 0.05 level (2-tailed); AI in DEP—Application of AI in health data extraction and processing (Question 2); Delegation on AI—Delegation of clinical procedures on AI tools (Question 3); Adv. of AI—Specific advantages of AI (Question 5); Disadv. of AI—Specific Disadvantages of using AI (Question 6); Pred. for using AI—Predisposition for using AI in clinical practice (Question 7); ICT use–use of information and communication technologies (Question 13); Com. of DT and AI—Self-perceived command of digital technologies and knowledge about AI (Question 14); YPE—Years of Professional Experience; Public—Public Sector; Private—Private Sector; PHC—Primary Health Care; HC—Hospital Care.
(DOCX)
