# Supplementary material for: Artificial intelligence in medicine: A comprehensive survey of medical doctor’s perspectives in Portugal
Source: PLoS One. 2023 Sep 7;18(9):e0290613. doi: 10.1371/journal.pone.0290613 (PMC10484446; doi:10.1371/journal.pone.0290613)
Supplement: S1 Table — Coefficients of correlation between AI perceptions (scores) and different study population characteristics. ns. = not significant; **Correlation is significant at the 0.01 level (2-tailed); * Correlation is significant at the 0.05 level (2-tailed); AI in DEP—Application of AI in health data extraction and processing (Question 2); Delegation on AI—Delegation of clinical procedures on AI tools (Question 3); Adv. of AI—Specific advantages of AI (Question 5); Disadv. of AI—Specific Disadvantages of using AI (Question 6); Pred. for using AI—Predisposition for using AI in clinical practice (Question 7); ICT use–use of information and communication technologies (Question 13); Com. of DT and AI—Self-perceived command of digital technologies and knowledge about AI (Question 14); YPE—Years of Professional Experience. (DOCX) [file pone.0290613.s001.docx]

**S1 Table - Correlation (Spearman) between different AI perceptions and study population characteristics.**

|  | AI in DEP | Delegation on AI | Adv. of AI | Disadv. of AI | Pred. for using AI | ICT use | Com. of DT and AI | Age | YPE |
| --- | --- | --- | --- | --- | --- | --- | --- | --- | --- |
| AI in DEP | 1 |  |  |  |  |  |  |  |  |
| Delegation on AI | 0,463** | 1 |  |  |  |  |  |  |  |
| Adv. of AI | 0,601** | 0,771** | 1 |  |  |  |  |  |  |
| Disadv. of AI | -0,384** | -0,276** | -0,327** | 1 |  |  |  |  |  |
| Pred. for using AI | 0,661** | 0,689** | 0,819** | -0,373** | 1 |  |  |  |  |
| ICT use | 0,252** | 0,170** | 0,204** | -0,202** | 0,263** | 1 |  |  |  |
| Com. of DT and AI | 0,158** | 0,096** | 0,141** | -0,101** | 0,139** | 0,171** | 1 |  |  |
| Age | ns. | 0,119** | ns. | ns. | ns. | -0,235** | -0,094** | 1 |  |
| YPE | ns. | 0,113** | ns. | ns. | ns. | -0,226** | -0,080* | 0,957** | 1 |
