# Supplementary material for: Artificial intelligence in medicine: A comprehensive survey of medical doctor’s perspectives in Portugal
Source: PLoS One. 2023 Sep 7;18(9):e0290613. doi: 10.1371/journal.pone.0290613 (PMC10484446; doi:10.1371/journal.pone.0290613)
Supplement: S2 Table — Description of score results according to gender (statistically significant associations). (DOCX) [file pone.0290613.s002.docx]

**S2 Table – Indicators of AI perceptions (scores) according to gender.**

|  |  | Women | | Men | |  |
| --- | --- | --- | --- | --- | --- | --- |
|  |  | N | % | N | % |  |
| Application of AI in health data extraction and processing (Question 2)  p<0,001 | Agree | 416 | 83,7 | 384 | 88,7 |  |
|  |  |  |  |  |  |  |
|  | Disagree | 81 | 16,3 | 49 | 11,3 |  |
|  |  |  |  |  |  |  |
| Delegation of clinical procedures on AI tools  (Question 3)  p<0,001 | Agree | 218 | 59,9 | 247 | 76,0 |  |
|  |  |  |  |  |  |  |
|  | Disagree | 146 | 40,1 | 78 | 24,0 |  |
|  |  |  |  |  |  |  |
| Specific advantages of AI (  Question 5)  p<0,001 | Agree | 275 | 73,1 | 298 | 85,1 |  |
|  |  |  |  |  |  |  |
|  | Disagree | 101 | 26,9 | 52 | 14,9 |  |
|  |  |  |  |  |  |  |
| Specific Disadvantages of using AI  (Question 6)  p<0,001 | Agree | 298 | 85,6 | 201 | 68,1 |  |
|  |  |  |  |  |  |  |
|  | Disagree | 50 | 14,4 | 94 | 31,9 |  |
|  |  |  |  |  |  |  |
| Predisposition for using AI in clinical practice  (Question 7)  p<0,001 | Agree | 256 | 68,4 | 287 | 81,8 |  |
|  |  |  |  |  |  |  |
|  | Disagree | 118 | 31,6 | 64 | 18,2 |  |
|  |  |  |  |  |  |  |
| Use of information and communication technologies  (Question 13)  p<0,001 | Yes | 300 | 88,8 | 253 | 87,8 |  |
|  |  |  |  |  |  |  |
|  | No | 38 | 11,2 | 35 | 12,2 |  |
|  |  |  |  |  |  |  |
|  |  |  |  |  |  |  |
| Command of digital technologies and AI  (Question 14)  p<0,635 | Yes | 319 | 69,8 | 329 | 80,0 |  |
|  |  |  |  |  |  |  |
|  | No | 138 | 30,2 | 82 | 20,0 |  |
|  |  |  |  |  |  |  |
|  |  |  |  |  |  |  |
