# Supplementary material for: Artificial intelligence in medicine: A comprehensive survey of medical doctor’s perspectives in Portugal
Source: PLoS One. 2023 Sep 7;18(9):e0290613. doi: 10.1371/journal.pone.0290613 (PMC10484446; doi:10.1371/journal.pone.0290613)
Supplement: S3 Table — Description of score results according to radiology and other medical specialties (statistically significant associations). (DOCX) [file pone.0290613.s003.docx]

**S2a Table – Indicators of AI perceptions (scores) according to medical specialties (radiology).**

|  | | | | | | | | Radiology | | | | | | Other medical specialties | | |  |
| --- | --- | --- | --- | --- | --- | --- | --- | --- | --- | --- | --- | --- | --- | --- | --- | --- | --- |
|  |  |  |  |  |  |  |  | N | | | | | Mean±SD | N | | Mean±SD |  |
| Application of AI in health data extraction and processing (Question 2)  p<0,099 | | | | | | | | 31 | | | | | 5,1±1.1 | 930 | | 4,8±1.3 |  |
|  |  |  |  |  |  |  |  |  |  |  |  |  |  |  |  |  |  |
|  |  |  |  |  |  |  |  |  |  |  |  |  |  |  |  |  |  |
| Delegation of clinical procedures on AI tools  (Question 3)  p<0,413 | | | | | | | | 31 | | | | | 4,0±0.9 | 927 | | 3,9±1.0 |  |
|  |  |  |  |  |  |  |  |  |  |  |  |  |  |  |  |  |  |
|  |  |  |  |  |  |  |  |  |  |  |  |  |  |  |  |  |  |
| Specific advantages of AI  (Question 5)  p<0,077 | | | | | | | | 30 | | | | | 4,6±0.9 | 915 | | 4,2±1.1 |  |
|  |  |  |  |  |  |  |  |  |  |  |  |  |  |  |  |  |  |
|  |  |  |  |  |  |  |  |  |  |  |  |  |  |  |  |  |  |
| Specific Disadvantages of using AI  (Question 6)  p<0,400 | | | | | | | | 28 | | | | | 4,3±1.0 | 842 | | 4,1±1.1 |  |
|  |  |  |  |  |  |  |  |  |  |  |  |  |  |  |  |  |  |
|  |  |  |  |  |  |  |  |  |  |  |  |  |  |  |  |  |  |
|  |  | | | | | |  | |  | | |  | |  |  | |  |
|  | Predisposition for using AI in clinical practice  (Question 7)  p<0,033 | | | | | |  | | 23 | | | 4,7±0.9 | | 879 | 4,1±1.2 | |  |
|  |  |  |  |  |  |  | | | |  |  |  |  |  |  |  |  |
| Use of information and communication technologies  (Question 13)  p<0,509 | | | | | | | | 32 | | | | 4,3±0.8 | | 871 | 4,1±1.1 | |  |
|  |  |  |  |  |  |  |  |  |  |  |  |  |  |  |  |  |  |
|  |  |  |  |  |  |  |  |  |  |  |  |  |  |  |  |  |  |
| Command of digital technologies and AI  (Question 14)  p<0,437 | | | | | | | | 32 | | | | 3,8±0.3 | | 973 | 3,8±0.4 | |  |
|  |  |  |  |  |  |  |  |  |  |  |  |  |  |  |  |  |  |
|  |  |  |  |  |  |  |  |  |  |  |  |  |  |  |  |  |  |
|  |  |  |  |  |  |  |  |  |  |  |  |  |  |  |  |  |  |
