# Supplementary material for: Artificial intelligence in medicine: A comprehensive survey of medical doctor’s perspectives in Portugal
Source: PLoS One. 2023 Sep 7;18(9):e0290613. doi: 10.1371/journal.pone.0290613 (PMC10484446; doi:10.1371/journal.pone.0290613)
Supplement: S4 Table — Description of general statistics related with the scores and their respective questions. (DOCX) [file pone.0290613.s004.docx]

**S3 Table – Descriptive statistics of the indicators of AI perceptions (scores).**

|  | Application of AI in health data extraction and processing (Question 2) | Delegation of clinical procedures on AI tools (Question 3) | Specific advantages of AI (Question 5) | Specific Disadvantages of using AI (Question 6) | Predisposition for using AI in clinical practice (Question 7) | Use of information and communication technologies (Question 13) | Self-perceived command of digital technologies and knowledge about AI (Question 14) |
| --- | --- | --- | --- | --- | --- | --- | --- |
| N (valid) | 961 | 958 | 945 | 870 | 902 | 1003 | 1005 |
| Missing values | 52 | 55 | 68 | 143 | 111 | 10 | 8 |
| Mean | 4,8044 | 3,8643 | 4,2038 | 4,0842 | 4,147 | 4,1142 | 3,76 |
| Median | 5 | 3,9 | 4,2222 | 4,1111 | 4,2857 | 4 | 4 |
| Std. deviation | 1,2687 | 1,04242 | 1,14098 | 1,05185 | 1,20856 | 1,10577 | 0,35147 |
| Minimum | 1 | 1 | 1 | 1 | 1 | 1 | 1,75 |
| Maximum | 6 | 6 | 6 | 6 | 6 | 6 | 4 |
