# Supplementary material for: Artificial intelligence in medicine: A comprehensive survey of medical doctor’s perspectives in Portugal
Source: PLoS One. 2023 Sep 7;18(9):e0290613. doi: 10.1371/journal.pone.0290613 (PMC10484446; doi:10.1371/journal.pone.0290613)
Supplement: S5 Table — Results of Cronbach´s Alpha test (questions 2, 3, 5, 6 and 7 of our survey) for measure of internal consistency (reliability test), and the respective coefficient´s level (Coefficient of Cronbach´s Alpha: more than 0.9—Excellent; 0.80–0.89 –Good; 0.70–0.79 –Acceptable; 0.6–0.69 –Questionable; 0.5–0.59 –Poor; less than 0.59 –Unacceptable). 1* Corrected total item correlation [62]. (DOCX) [file pone.0290613.s005.docx]

S4 Table - Internal consistency of the questionnaire.

| **Internal Consistency** | | |
| --- | --- | --- |
|  | 1* | α |
| **Question 2: Regarding data extraction and processing, please rate your degree of agreement with the following statements:** | | |
| Intelligent data extraction and processing via AI tools can improve the quality of healthcare delivery in general. | 0,918 | 0,982 |
| Intelligent data extraction and processing via AI tools can improve the quality of healthcare delivery in the area of your specialty. | 0,923 | 0,981 |
| Total coefficient of the variable (α) | 0,975 | |
| **Question 3: Between the following procedures, please indicate the degree of agreement that this can be delegated on an Artificial Intelligence tool:** | | |
| Evaluate cardiac frequency | 0,497 | 0,894 |
| Collect medical history | 0,56 | 0,891 |
| Ask and systematize general symptomatology | 0,646 | 0,886 |
| Evaluate blood pressure | 0,479 | 0,894 |
| Prescribe medical exams | 0,681 | 0,883 |
| Report imaging exams | 0,645 | 0,886 |
| Perform differential diagnosis based on symptoms and medical exams | 0,709 | 0,882 |
| Recommend therapeutic strategies based on a diagnosis validated by a medical doctor | 0,62 | 0,887 |
| Renew previous prescriptions of medical doctors | 0,646 | 0,885 |
| Recommend lifestyle changes (diet, physical activity) based on symptoms and the result of medical exams | 0,546 | 0,893 |
| Total coefficient of the variable (α) | 0,896 | |
| **Question 5: Please indicate your degree of agreement on the following possible advantages of using Artificial Intelligence tools in healthcare:** | | |
| Simplification and facilitation of patient care. | 0,691 | 0,929 |
| Facilitation of medical care in populations that are isolated or have difficulties in accessing the health system. | 0,652 | 0,931 |
| Increased accuracy in collecting patient clinical history. | 0,711 | 0,929 |
| Increased adequacy of prescribing complementary means of diagnosis. | 0,781 | 0,925 |
| Increased diagnostic accuracy. | 0,816 | 0,923 |
| Increased accuracy in therapeutic prescription. | 0,792 | 0,924 |
| Performance of routine tasks, freeing up medical professionals for other tasks. | 0,673 | 0,93 |
| Boost the storage of health information and facilitating access to it. | 0,584 | 0,934 |
| Decrease in medical errors. | 0,748 | 0,926 |
| Total coefficient of the variable (α) | 0,934 | |
| **Question 6: Please indicate your degree of agreement on the following possible disadvantages of using AI tools in healthcare:** | | |
| Increased dehumanization of healthcare. | 0,657 | 0,875 |
| Potentiation of the resentment of health professionals because they fear being replaced. | 0,428 | 0,892 |
| Potentiation of distance from patients due to low levels of health and digital literacy. | 0,632 | 0,877 |
| Decreased ability to improvise in care provision. | 0,543 | 0,883 |
| Decreased quality of healthcare provided. | 0,656 | 0,875 |
| Uncertainty about the risks that this type of technology can bring. | 0,723 | 0,871 |
| Uncertainty about the benefits that this type of technology can bring. | 0,663 | 0,875 |
| Increased risk of invasion of users' privacy and violations of health information security. | 0,56 | 0,883 |
| Threatens the sustainability of health systems due to the high investment required to implement this type of technology. | 0,556 | 0,883 |
| Total coefficient of the variable (α) | 0,889 | |
| **Question 7: Please indicate your degree of agreement with the following statements regarding the use of Artificial Intelligence in your practice:** | | |
| It would be easy to include the use of AI in my clinical practice. | 0,644 | 0,935 |
| It would be useful to include the use of AI in my clinical practice. | 0,836 | 0,921 |
| I would use AI within my individual professional activity. | 0,858 | 0,919 |
| I would use AI to assist me in collecting the medical history. | 0,66 | 0,936 |
| I would use AI to help me perform a diagnosis. | 0,806 | 0,923 |
| I would use AI to help me define a therapeutic prescription. | 0,798 | 0,923 |
| I would use AI to assist me in the daily management of my professional activity. | 0,642 | 0,934 |
| Total coefficient of the variable (α) | 0,934 | |
