# Supplementary material for: Artificial intelligence in medicine: A comprehensive survey of medical doctor’s perspectives in Portugal
Source: PLoS One. 2023 Sep 7;18(9):e0290613. doi: 10.1371/journal.pone.0290613 (PMC10484446; doi:10.1371/journal.pone.0290613)
Supplement: S6 Table — U-test values for statistically significant associations between AI perceptions (scores) and different study population characteristics. ns. = not significant; ** Significant at the 0.01 level (2-tailed); * Significant at the 0.05 level (2-tailed); AI in DEP—Application of AI in health data extraction and processing (Question 2); Delegation on AI—Delegation of clinical procedures on AI tools (Question 3); Adv. of AI—Specific advantages of AI (Question 5); Disadv. of AI—Specific Disadvantages of using AI (Question 6); Pred. for using AI—Predisposition for using AI in clinical practice (Question 7); ICT use–use of information and communication technologies (Question 13); Com. of DT and AI—Self-perceived command of digital technologies and knowledge about AI (Question 14); YPE—Years of Professional Experience; Public—Public Sector; Private—Private Sector; PHC—Primary Health Care; HC—Hospital Care. (DOCX) [file pone.0290613.s006.docx]

**S5 Table - Associations between different AI perceptions and study population characteristics (Mann-Whitney U).**

|  | AI in DEP | Delegation on AI | Adv. of AI | Disadv. of AI | Pred. for using AI | ICT use | Com. of DT and AI |
| --- | --- | --- | --- | --- | --- | --- | --- |
| Gender | 90715** | 90160,5** | 88658,5** | 72930,5** | 76115,5** | 96535,5** | ns. |
| Public | ns. | ns. | ns. | ns. | ns. | ns. | ns. |
| Private | ns. | ns. | ns. | ns. | ns. | ns. | ns. |
| PHC | ns. | ns. | ns. | ns. | ns. | ns. | ns. |
| HC | ns. | 95265,500* | ns. | ns. | ns. | ns. | ns. |
